# New Books

**Published:** 2009-03

**Air Quality and Ecological Impacts: Relating Sources to Effects**

Allan Legge, ed.

St. Louis, MO:Elsevier, 2009. 322 pp. ISBN: 978-0-08-095201-7, $186

**Beyond “Fortress America”: National Security Controls on Science and Technology in a Globalized World**

Committee on Science, Security, and Prosperity; Committee on Scientific Communication and National Security; National Research Council

Washington, DC:National Academies Press, 2009. 170 pp. ISBN: 978-0-309-13066-0, $41.95

**Bioinformatics: Applications in Life and Environmental Sciences**

M.H. Fulekar, ed.

New York:Springer, 2009. 246 pp. ISBN: 978-1-4020-8879-7, $159

**Biological Science for the Biomedical and Healthcare Sciences**

Gillian Pocock, Chris Richards

New York:Oxford University Press, 2009. 480 pp. ISBN: 978-0-19-928907-3, $65

**Blue Covenant: The Global Water Crisis and the Coming Battle for the Right to Water**

Maude Barlow

New York:New Press, 2009. 208 pp. ISBN: 978-1-59558-453-3, $16.95

**Climate Change: Observed Impacts on Planet Earth**

Trevor Letcher, ed.

St. Louis, MO:Elsevier, 2009. 460 pp. ISBN: 978-0-444-53301-2, $90

**Environmental Health Sciences Decision Making: Risk Management, Evidence, and Ethics**

Yank Coble, Christine Coussens, Kathleen Quinn, eds.

Washington, DC:National Academies Press, 2009. 92 pp. ISBN: 978-0-309-12454-6, $21

**Epigenomics**

Anne C. Ferguson-Smith, John M. Greally, Rob A.Martienssen, eds.

New York:Springer, 2009. 442 pp., ISBN: 978-1-4020-9186-5, $219

**Genomics and Environmental Regulation**

Ricard R. Sharp, Gary E. Marchant, Jamie A. Grodsky, eds.

Baltimore, MD:Johns Hopkins University Press, 2008. 353 pp. ISBN: 978-0-8018-9022-2, $50

**Handbook of Toxicology of Chemical Warfare Agents**

Ramesh Gupta, ed.

St. Louis, MO:Elsevier, 2009. 1,200 pp. ISBN: 978-0-12-374484-5, $225

**Introduction to Food Toxicology, 2nd ed.**

Takayuki Shibamoto, Leonard Bjeldanes

St. Louis, MO:Elsevier, 2009. 368 pp. ISBN: 978-0-12-374286-5, $79.95

**Pharma-Ecology**

Patrick K. Jjemba

Hoboken, NJ:John Wiley & Sons, Inc., 2008. 250 pp. ISBN: 978-0-470-04630-2, $95

**Politics of China’s Environmental Protection: Problems and Progress**

Chen Gang

Hackensack, NJ:World Scientific Publishing, Inc., 2009. 300 pp. ISBN: 978-981-283-869-8, $78

**State of the World’s Oceans**

M. Allsopp, R. Page, P. Johnston, D. Santillo

New York:Springer, 2009. 258 pp. ISBN: 978-1-4020-9115-5, $79.95

**Statistical Methods for Environmental Epidemiology with R: A Case Study in Air Pollution and Health**

Roger D. Peng, Francesca Dominici

New York:Springer, 2008. 144 pp. ISBN: 978-0-387-78166-2, $49.95

**Sustaining Life: How Human Life Depends on Biodiversity**

Eric Chivian, Aaron Bernstein

New York:Oxford University Press, 2008. 576 pp. ISBN: 978-0-19-517509-7, $34.95

**The Great Barrier Reef: Biology, Environment and Management**

Pat Hutchings, Mike Kingsford, Ove Hoegh-Guldberg, eds.

New York:Springer, 2009. 392 pp. ISBN: 978-1-4020-8949-7, $119

**Water, Place, & Equity**

John M. Whiteley, Helen Ingram, Richard Warren Perry, eds.

Cambridge, MA:MIT Press, 2008. 312 pp. ISBN: 978-0-262-73191-1, $25

